# Clinicopathological features of tumor mutation burden, Epstein-Barr virus infection, microsatellite instability and PD-L1 status in Chinese patients with gastric cancer

**DOI:** 10.1186/s13000-021-01099-y

**Published:** 2021-05-01

**Authors:** Li Zhang, Yinkui Wang, Zhongwu Li, Dongmei Lin, Yiqiang Liu, Linxin Zhou, Dongliang Wang, Aiwen Wu, Ziyu Li

**Affiliations:** 1 Department of Pathology, Key Laboratory of Carcinogenesis and Translational Research, Peking University Cancer Hospital, No.52 Fucheng Road Haidian District, Beijing, 100142 People’s Republic of China; 2 Department of Gastrointestinal Surgery, Key Laboratory of Carcinogenesis and Translational Research, Peking University Cancer Hospital, No.52 Fucheng Road Haidian District, Beijing, 100142 People’s Republic of China; 3 ChosenMed, Beijing Economic-Technological Development Area, Beijing, 100176 People’s Republic of China

**Keywords:** Gastric cancer, MSI, Next generation sequencing, TMB, EBV-encoded RNA, PD-L1

## Abstract

**Objectives:**

Gastric cancer (GC) is the 4th most common type of cancer worldwide. Different GC subtypes have unique molecular features that may have different therapeutic methods. The aim of the present study was to investigate Epstein-Barr virus (EBV) infection, microsatellite instability (MSI) status, the expression of programmed death-ligand 1 (PD-L1) and gene mutations in GC patients.

**Methods:**

The data of 2504 GC patients, who underwent curative gastrectomy with lymphadenectomy at Peking University Cancer Hospital between 2013 and 2018, were reviewed. We analyzed the clinicopathological factors associated with the immunohistochemistry (IHC) profiles of these patients, and genetic alterations were analyzed using next generation sequencing (NGS).

**Results:**

Mismatch repair-deficient (d-MMR) GC patients were found to have a higher probability of expressing PD-L1 (*p* = 0.000, PD-L1 cutoff value = 1%). In addition, 4 and 6.9% of the 2504 gastric cancer patients were EBV-positive and d-MMR, respectively. The number of MLH1/PMS2-negative cases was 126 (6%), and the number of MSH2/MSH6-negative cases was 14 (0.9%). d-MMR status was associated with a intestinal group (*p* = 0.012), but not with tumor differentiation. Furthermore, MSI and d-MMR GC status (detected by NGS and IHC, respectively) were consistently high, and the rate of MSI was higher in patients with d-MMR GC. A number of genes associated with DNA damage repair were detected in GC patients with MSI, including POLE, ETV6, BRCA and RNF43. In patients with a high tumor mutation burden, the most significantly mutated genes were LRP1B (79.07%), ARID1A (74.42%), RNF43 (69.77%), ZFHX3 (65.12%), TP53 (58.14%), GANS (51.16%), BRCA2 (51.16%), PIK3CA (51.16%), NOTCH1 (51.16%), SMARCA4 (48.84%), ATR (46.51%), POLE (41.86%) and ATM (39.53%).

**Conclusions:**

Using IHC and NGS, MSI status, protein expression, tumor mutation burden (TMB) and genetic alterations were identified in patients with GC, which provides a theoretical basis for the future clinical treatment of GC.

## Introduction

Gastric cancer (GC) is one of the most commonly cancers, which has high mortality worldwide [1]. Different molecular alterations led to the identification of distinct GC subtypes. GC was divided into four molecular subtypes, which were EBV infection subtype, microsatellite instability (MSI) type, genome stable type and chromosome unstable type [2, 3]. These different subtypes could be used to guide therapeutic practice. The new classification is helpful to the selection of targeted drugs for gastric cancer patients. EBV infection can induce gene hypermethylation and tumorigenesis.

Immune checkpoint inhibitors can be used for EBV positive patients and MSI patients [4–8]. Currently, the expression of programmed death-ligand 1 (PD-L1) can be used as a marker of immunotherapy [9]. EBV and MSI GC subgroups may benefit from immunotherapy with PD-1/PD-L1 antibodies [10, 11]. Immune checkpoint inhibitors may also enhance antitumor activity in advanced GC patients [12–15].

Human epidermal growth factor receptor-2 (HER-2) is proto-oncogene, which is a transmembrane tyrosine kinase receptor. It is a key driver of GC tumorigenesis, which regulate cellular proliferation, apoptosis and differentiation. Trastuzumab is a therapeutic option for HER-2-positive patients [16, 17]. The aim of the present study was to investigate EBV infection status using in situ hybridization (ISH), and to determine mismatch repair (MMR) status, PD-L1 and HER-2 expression using immunohistochemistry (IHC), in surgically treated GC patients. Additionally, we analyzed the clinicopathological factors associated with these IHC profiles, and used next generation sequencing (NGS) technology to analyze the gene alterations and tumor mutation burden (TMB) of patients with GC.

## Materials and methods

### Patients and general information

In the present study, we reviewed all gastric adenocarcinoma patients who underwent curative gastrectomy between 2013 and 2018. Surgical specimens were fixed in 10% buffered formalin. GC TNM staging was conducted according to AJCC 8th TNM staging system. The study was approved by the ethics committee of Peking University Cancer Hospital, and all patients provided written informed consent prior to surgery.

### Immunohistochemical evaluation of PD-L1 expression

All paraffin-embedded specimens were cut into 4-μm sections. IHC staining was performed according to the manufacturer’s protocol. All tissue slices were evaluated by two pathologists. Specimens were scored based on the area of positively stained tumor cells or tumor-infiltrating immune cells as follows: 1, Positive staining area < 1%; 2, from 1 to < 10% positive staining; 3, from 10 to < 50% positive staining; or 4, ≥50% positive staining. The primary antibody against PD-L1 (SP142) was purchased from Spring Bioscience (Pleasanton, CA, USA).

### Evaluation of MMR protein expression and EBV infection status by IHC and ISH

Tumors were considered to have lost MLH1, MSH2, PMS2 or MSH6 expression only if there was a complete absence of nuclear staining in tumor cells; normal epithelial cells and lymphocytes were used as the internal controls. MMR protein expression was assessed by IHC using antibodies against the following: MLH1 (clone no. GM002); MSH2 (clone no. RED2); MSH6 (clone no. EP49); and PMS2 (clone no. EP51) (all Gene Tech Biotechnology Co., Ltd., Shanghai, China). The complete absence of protein expression (0+ in 100% of cells) was considered to indicate the loss of MMR, and thus d-MMR. An EBV-encoded RNA (EBER) ISH kit (OriGene Technologies, Inc., Beijing, China) was used to determine EBV infection status, according to the manufacturer’s protocol.

### TMB and gene mutation analysis

NGS technology was used to detect the MSI status of the GC samples, including TMB and gene mutations (ChosenMed, Inc., Beijing, China). TMB was assessed using the NGS platform (Illumina sequencing platform, PE150) with a sequencing depth greater than 3500x. The candidate MSI loci were detected by identifying a sequence of 1–5 bases with mutations that had repeated at least 5 times in the Bam file. The MSI threshold was determined according to large data sets from the European Genome-phenome Archive and TCGA panels: < 20% was considered to be microsatellite stable (MSS), 20–30% indicated MSI-L and > 30% was considered as MSI-H. Gene mutations were obtained using an assembly clustering algorithm, not by simple cutoff values; the detection limit of the tissue samples was 2%. The variation in the normal samples was ‘SNP’, and the specific variation of the tumor samples was ‘somatic mutation’. Somatic mutations include synonymous mutation, missense mutation and nonsense mutation.

### Immunohistochemical detection of HER-2 expression

Tissues were stained and scored according to the HER-2 Detection Guide for Gastric Cancer as follows: 0, < 10% tumor cell membrane staining; 1+, ≥10% tumor cell membrane staining, weak or faintly visible membrane staining, or only partial membrane staining; 2+, ≥10% tumor cells with weak to moderate basal membrane, lateral membrane or complete membrane staining; and 3+, ≥10% strong tumor cell basal membrane, lateral membrane or complete membrane staining.

### Statistical analysis

Comparisons between categorical variables were conducted using the χ^2^ test or Fisher’s exact test as appropriate. Differences in *p*-values < 0.05 were considered to be statistically significant.

## Results

### Association between PD-L1 expression and the clinicopathological features of GC

**Fig. 1:**
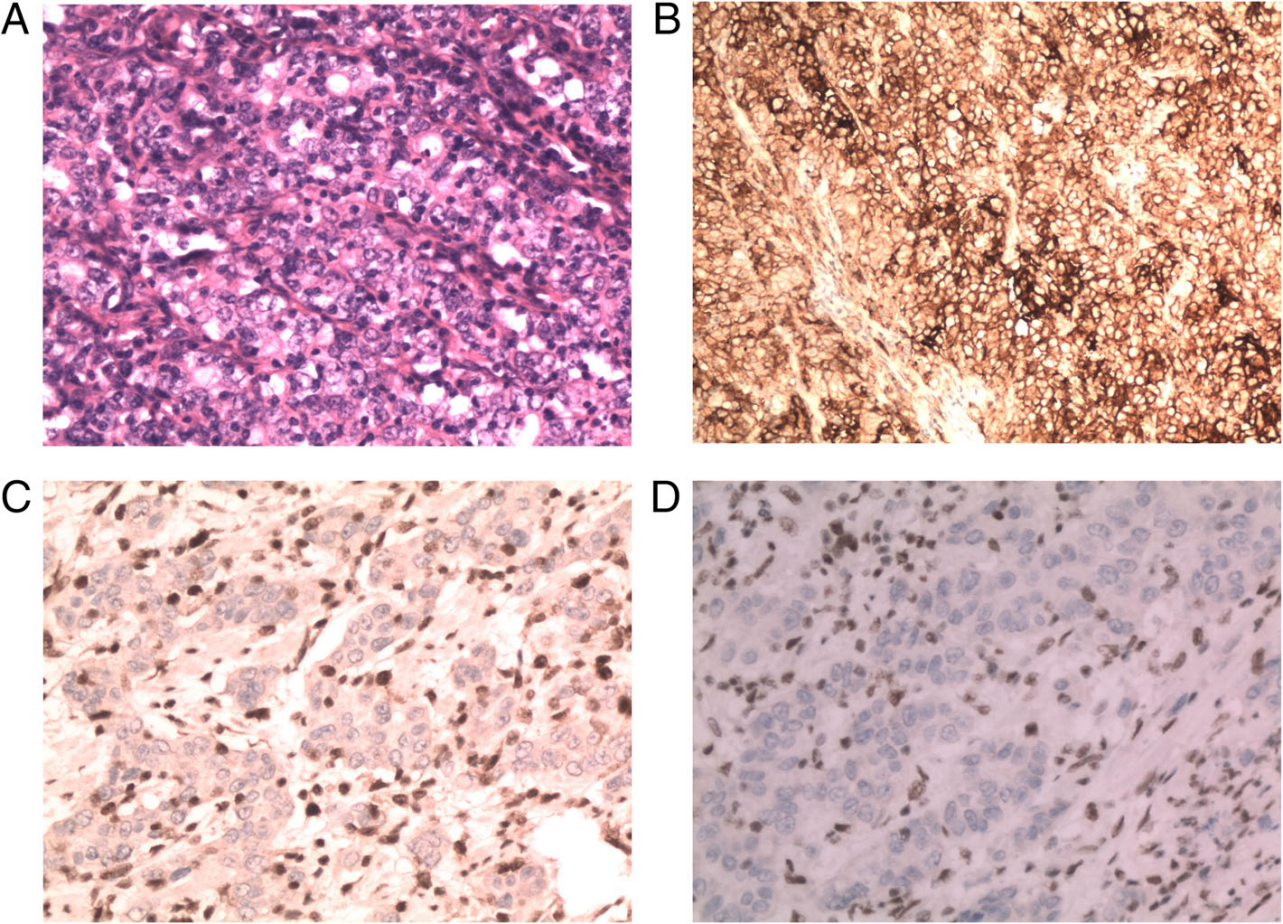
Strong PD-L1 staining in patients with d-MMR GC. **a** Poorly differentiated GC; H&E staining, × 200 magnification. **b** The area of positive PD-L1 staining in tumor cells was > 90% (moderate- to strong-positive); × 200 magnification. **c** MLH1 expression-negative IHC staining, × 200 magnification; stromal cells with positive staining were used as the internal control; **d** PMS2 expression-negative IHC staining, × 200 magnification; stromal cells were used as internal positive control. PD-L1, programmed death-ligand 1; d-MMR, mismatch repair-deficient; GC, gastric cancer; H&E, hematoxylin and eosin; IHC, immunohistochemical

PD-L1-positive cases were defined by the presence of membrane staining in least 1% of tumor cells or tumor-infiltrating immune cells. Accordingly, the proportion of PD-L1-positive cases accounted for 20.2% of the patients investigated. Tumor cell PD-L1 expression was identified in 11.6, 10.9 and 4% of cases, at different cut-off points, respectively (1, 10, and 50%, according to the positively stained area of the cell membrane). d-MMR GC patients were found to be more likely to express PD-L1 than p-MMR patients (*p* = 0.000; PD-L1 cutoff value = 1%) (Fig. 1).

### MMR protein expression status and clinicopathological features

In total, 140 of the 2031 cases (6.9%) were d-MMR. The number of MLH1/PMS2 protein deficient cases was 126/2031 (6.2%), and the number of MSH2/MSH6 deficient patients was 14/2031 (0.7%). d-MMR status was associated with intestinal group (*p* = 0.012), but not with tumor differentiation (*p* = 0.256) (Table 1).

**Table 1:** Clinicopathological features of 2031 p-MMR and d-MMR GC patients

Variables	MMR status		*p* value
	p-MMR	d-MMR	
Sex			0.102
male	516/2031 (25.4%)	55/2031 (2.7%)	
female	1375/2031 (67.7%)	85/2031 (4.2%)	
Lauren type			0.012*
Diffuse/mixed	1201/2031 (59.1%)	74/2031 (3.6%)	
intestinal	690/2031 (34%)	66/2031 (3.2%)	
Differentiation			0.256
poorly	1264/2031 (62.2%)	87/2031 (4.3%)	
well-moderately	627/2031 (30.9%)	53/2031 (2.6%)	
T stage			0.038
pT3 + T4	1193/2031 (58.7%)	88/2031 (4.3%)	
pT1 + T2	698/2031 (34.4%)	52/2031 (2.6%)	
Lymphnode metastasis			0.246
LNM+	1121/2031 (55.2%)	76/2031 (3.7%)	
LNM-	770/2031 (37.9%)	64/2031 (3.2%)	
PD-L1 expression			0.000*
PD-L1 -	1337/2031 (65.8%)	69/2031 (3.4%)	
PD-L1 > =1%	554/2031 (27.3%)	71/2031 (3.5%)	

### EBER ISH ratio in patients with GC

**Fig. 2:**
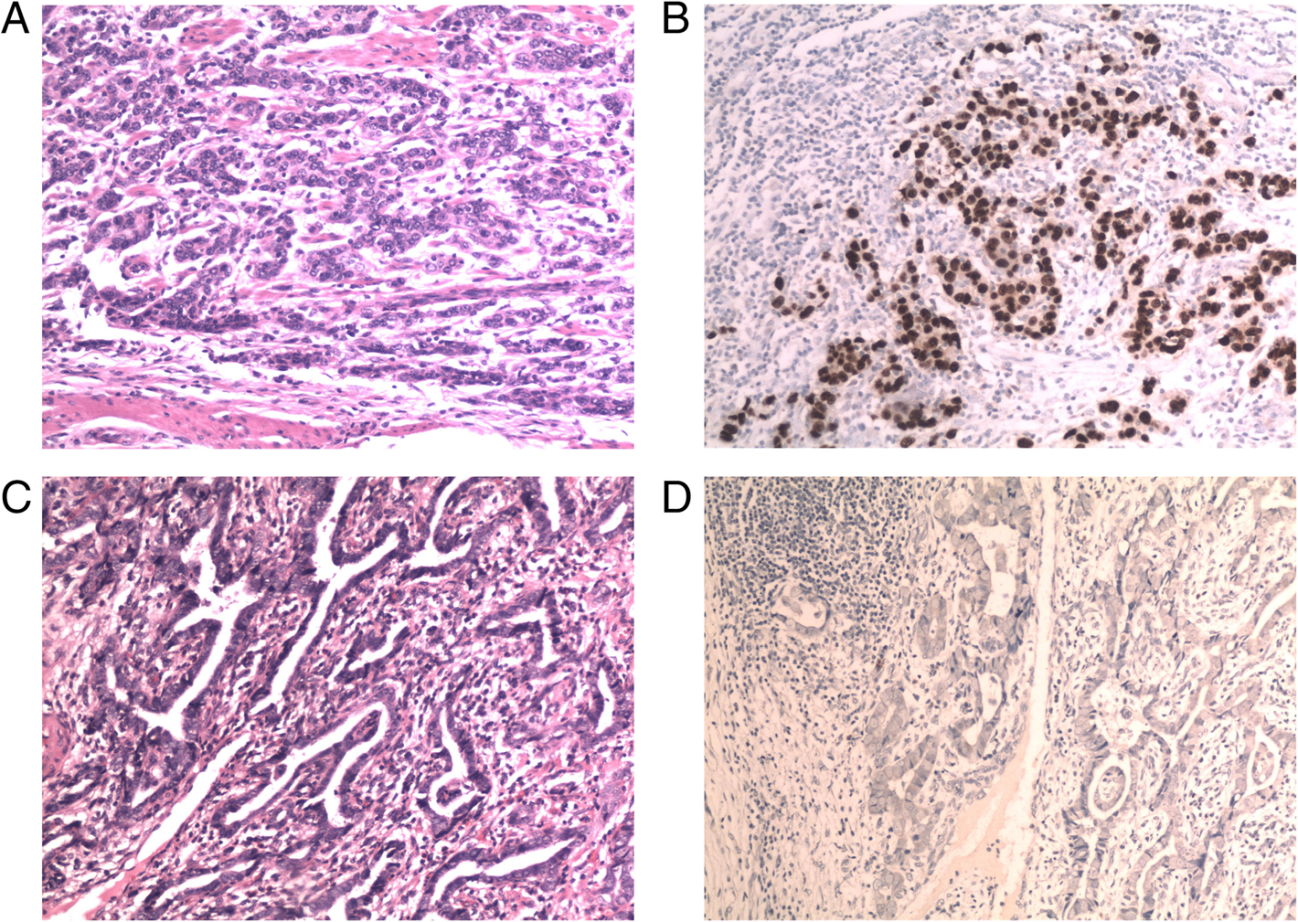
EBER-positive patients primarily exhibit diffused/mixed Lauren type and poor tumor differentiation. **a** Poorly differentiated GC, diffuse type; H&E staining, × 200 magnification. **b** Poorly differentiated GC; EBER ISH-positive staining, 200x magnification. **c** Moderately differentiated GC, intestinal type; H&E staining, × 200 magnification. **d** Moderately differentiated GC, EBER ISH-negative staining, 200x magnification. EBER, EBV-encoded RNA; GC, gastric cancer; H&E, hematoxylin and eosin; ISH, in situ hybridization

Of the 2504 patients investigated, 96 cases (4%) were EBV-positive and 2408 (96%) were EBER-negative. EBER-positive patients were predominantly male, with diffused/mixed Lauren type and poorly differentiated tumors (*p* < 0.05) (Table 2, Fig. 2). The level of PD-L1 expression was not significantly different between EBER-positive and EBER-negative patients (*p* = 0.524, PD-L1 cut off value = 1%).

**Table 2:** PD-L1 expression, EBER ISH status with clinic-pathological features of GC patients

Variables	EBER ISH status		*p* value
	EBER positive (+)	EBER negative (−)	
Sex			0.003*
male	82/2504 (3.3%)	1717/2504 (68.6%)	
female	14/2504 (0.6%)	691/2504 (27.5%)	
Lauren type			0.000*
Diffuse/mixed	77/2504 (3.1%)	1503/2504 (60%)	
intestinal	19/2504 (0.8%)	905/2504 (36.1%)	
Differentiation			0.001*
poorly differentiated	79/2504 (3.1%)	1600/2504 (63.9%)	
well-moderately	17/2504 (0.7%)	808/2504 (32.3%)	
T stage			0.322
pT3 + T4	55/2504 (2.2%)	1500/2504 (59.9%)	
pT1 + T2	41/2504 (1.6%)	908/2504 (36.3%)	
Lymphnode metastasis			0.326
LNM+	53/2504 (2.1%)	1450/2504 (57.9%)	
LNM-	43/2504 (1.7%)	958/2504 (38.3%)	
PD-L1 expression			0.524
PD-L1 -	52/2358 (2.2%)	1617/2358 (68.6%)	
PD-L1 > =1%	25/2358 (1.1%)	664/2358 (28.1%)

### The association between HER-2 expression, MMR status and EBER status

In the present study, the number of HER-2 1+ patients was 628/2504 (25.1%), the number of those with HER-2 2+ staining was 313/2504 (12.5%), and 102/2504 patients (4.1%) were HER-2 3+. There were 1461 patients without HER-2 protein expression, and the ratio of positive-to-negative expression was 58.3%. HER-2 expression was not found to be associated with MMR or EBER status (*p* = 0.129 and *p* = 0.300, Tables 3 and 4, respectively).

**Table 3:** Correlation of HER-2 expression and EBER status in GC patients

Variables	EBER+	EBER-	*p* value
HER-2 3+	2/3403 (0.6%)	102/3403 (3%)	0.300
HER-2 0/1+/2+	94/3403 (2.8%)	3205/3403 (94.2%)

**Table 4:** Correlation of HER-2 expression and MMR status in 2031 GC patients

Variables	p-MMR	d-MMR	*p* value
HER-2 3+	75/2031 (3.7%)	2/2031 (0.1%)	0.129
HER-2 0/1+/2+	1816/2031 (89.4%)	138/2031 (6.8%)	

### Results of NGS, and comparison of NGS-MSI and IHC-MMR results in GC

**Fig. 3:**
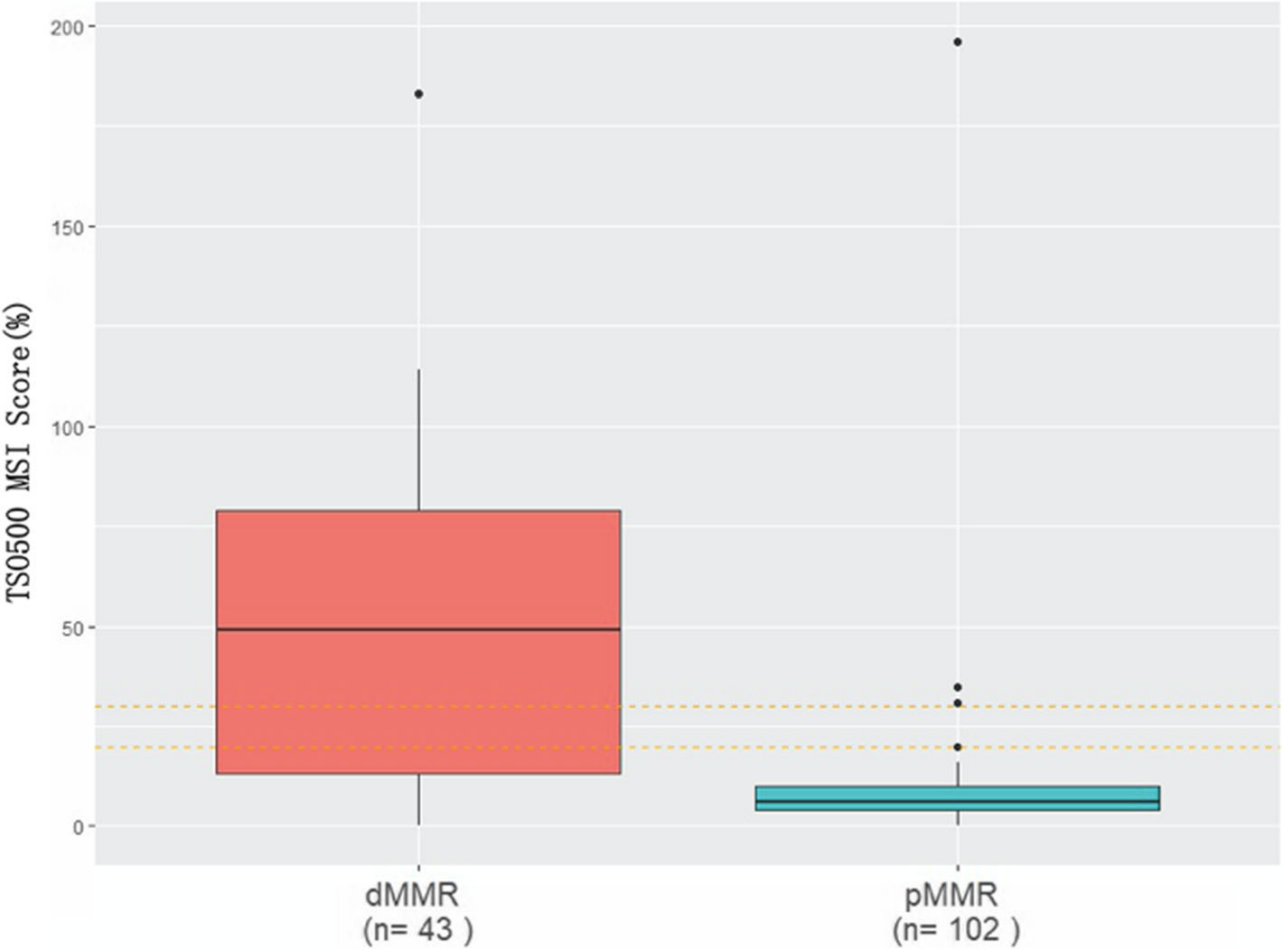
TSO500 MSI scores of patients with d-MMR GC are higher than those with p-MMR GC. The results of MSI detection (by next generation sequencing) were highly consistent with the immunohistochemistry results of those with d-MMR GC. d-MMR patients had higher MSI scores. MSI, microsatellite instability; d-MMR, mismatch repair-deficient; p-MMR, mismatch repair-proficient; GC, gastric cancer

**Fig. 4:**
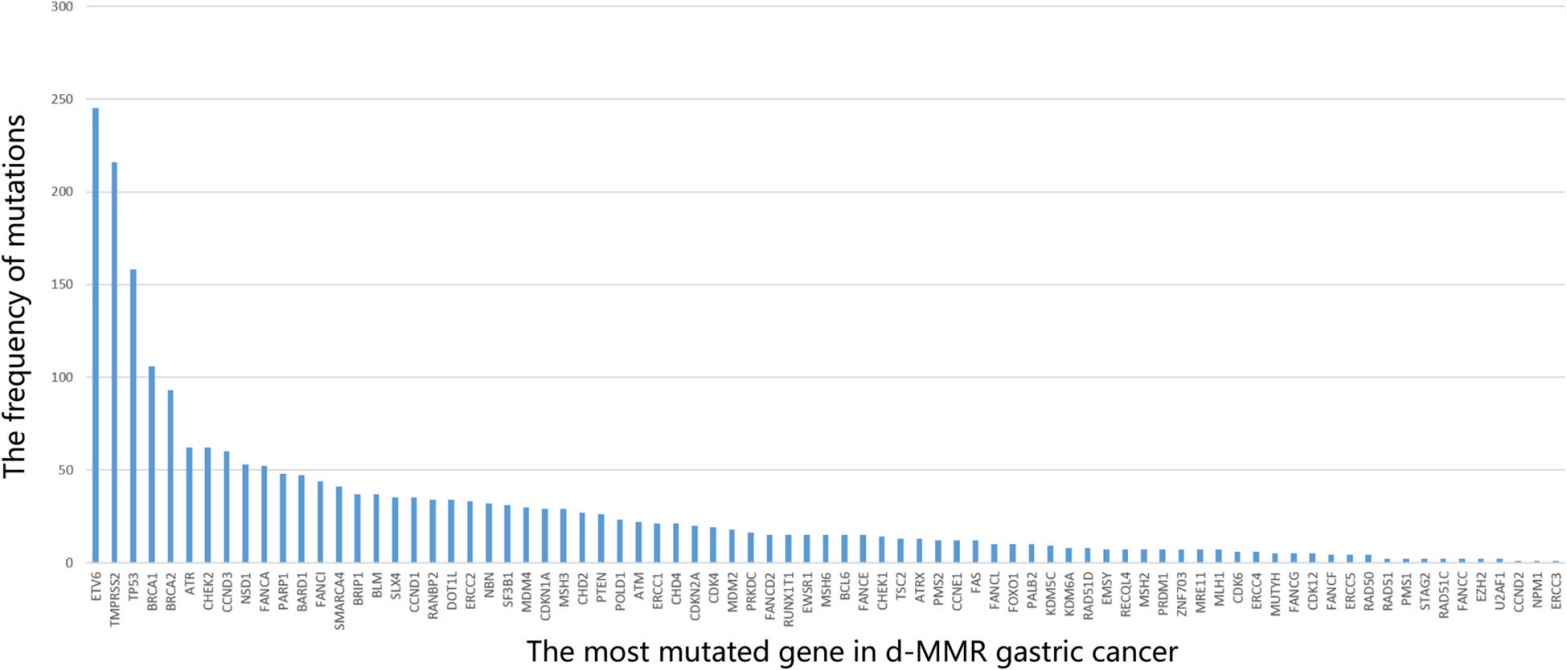
Overview of the frequency of DDR gene mutations in d-MMR GC samples. Various DDR genes were detected, including POLE, ETV6, ATR, TMPRS52, BRCA1, BRCA2, CHEK2, CCND3, FANCA and NSD1. DDR, DNA damage repair; d-MMR, mismatch repair-deficient; GC, gastric cancer

**Fig. 5:**
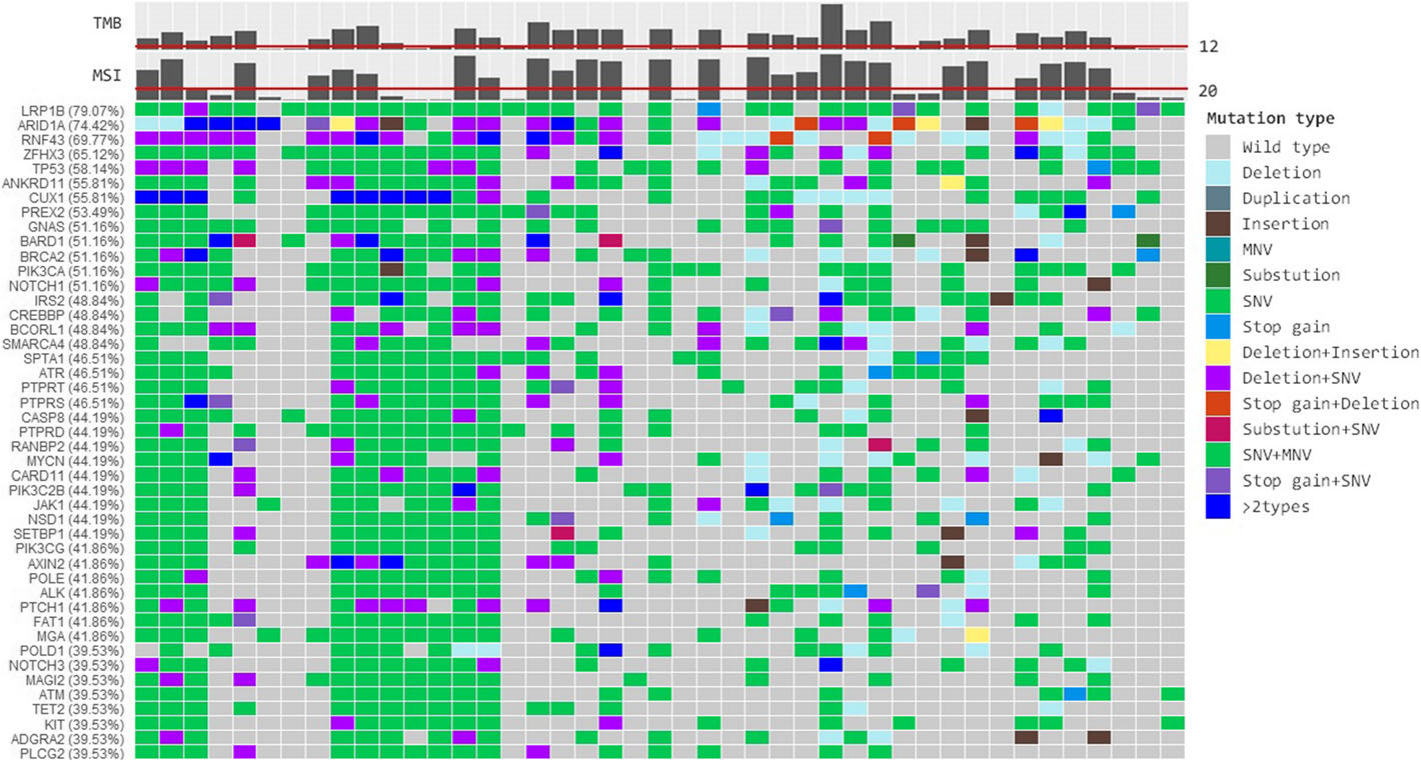
Oncoplot reporting the most recurrently mutated genes across the d-MMR GC samples analyzed in this study. TMB, MSI status, gene mutation frequency and mutation type were detected by next generation sequencing. In high-TMB and MSI tumors, the deletion mutation of certain genes (such as RNF43, BCORL1 and ATR) is apparent. d-MMR, mismatch repair-deficient; GC, gastric cancer; TMB, tumor mutation burden; MSI, microsatellite instability

We selected 43 d-MMR cases for NGS detection. The MSI (detected by NGS) and IHC results of patient with d-MMR GC were highly consistent; patients with d-MMR status had higher MSI scores, while those with p-MMR GC possessed comparatively lower scores (Fig. 3). A number of genes associated with DNA damage repair (DDR) were detected in MSI patients, such as ETV6, TP53, BRCA, ATR, FANCA, BARD1, POLE and RNF43 (Fig. 4). In GC patients with a high TMB, the most significantly mutated genes were LRP1B (79.07%), ARID1A (74.42%), RNF43(69.77%), ZFHX3(65.12%), TP53(58.14%), GANS (51.16%), BRCA2(51.16%), PIK3CA (51.16%), NOTCH1 (51.16%), SMARCA4 (48.84%), ATR (46.51%), POLE (41.86%) and ATM (39.53%) (Fig. 5).

## Discussion

GC is the fourth most common type of cancer worldwide. It is one of most lethal cancers, and it is a heterogeneous disease. Because of late diagnosis, the disease is often inoperable, and often recurs after resection. Systemic chemotherapy may be the only method for unresectable advanced gastric cancer [18]. Traditionally, GC classification has been based on histopathological and morphological features, which were first described in 1965 [19, 20]. But this classification was unable to identify molecular targets. It is important to select prognostic and predictive biomarkers in GC. This can be as a guide for GC precision medicine treatment. Large scale molecular profiling via NGS has resulted in molecular-based classification systems, which was helpful for targeted therapy. HER-2 overexpression is an important predictive indicator in GC, however, no large-scale studies on HER-2 expression in GC have been conducted in China. Wang et al [21] studied 135 patients with GC, where the expression rate of HER-2 protein was 39.3%. In our study, 2504 GC patients were analyzed, among whom positive cases patients with HER-2 protein (3+) accounted for 4.1%, and patients with HER-2 protein (2+) expression accounted for 12.5%. HER-2 expression was not found to be associated with EBER or MMR status, nor was it related to MMR status. However, in our previous study of > 3000 cases of colorectal cancer, HER-2 3+ positive expression was found to be more prevalent in p-MMR patients. PD-L1 can be used as a marker of immunotherapy, which is important in clinical treatment. Anti-PD-1/PD-L1 immunotherapy may be used to treat patients with HER-2-negative GC, which provides an alternative treatment option for these individuals. A number of studies have demonstrated that GC patients with EBV infection comprise ~ 9% of all cases of GC, and the patients can be treated with immunotherapy [22]. In our study, EBV positivity was 4%, and the EBV-positive patients were predominantly male, with a diffused/mixed Lauren type and poor tumor differentiation (*p* < 0.001). The EBV infection rate in our study was also lower than the global average. The Alaska Native (AN) population exhibit the highest incidence and mortality rates of GC in North America, with an EBV infection rate of 20%, which is far greater than the global average of 10% [23]. In a Japanese study of 1067 GC cases, the positive rate of EBV infection was 7.1% [24], indicating that the EBV infection rate of GC differs between regions. In contrast to other GC subtypes, GC patients with EBV infection exhibited a number of distinct characteristics in the present study. With a PD-L1 positivity rate > 1%, there was no significant difference in the level of PD-L1 expression between EBV-positive and EBV-negative patients (*p* = 0.524). In a small case study [11], PD-L1 expression was significantly associated with EBV infection (*p* < 0.001). In our study of 2504 patients, high expression levels of PD-L1 were more likely to occur in d-MMR patients (*p* < 0.001; PD-L1 cutoff value = 1%). In a study by Haron et al [25], a total of 60 GC cases were retrieved. Microsatellite analysis identified 10 MSI-positive cases (16.7%), of which six (10.3%) did not express MLH1 (*n* = 3) or MSH2 (n = 3) protein. In our study, the number of MLH1/PMS2 protein deficient cases was 126 (6%), and the number of MSH2/MSH6 protein deficient cases was 14 (0.9%). Furthermore, d-MMR GC patients were more likely to express PD-L1 (*p* < 0.001). We think that different types of PD-L1 antibodies, different tissue processing methods, and different systems for evaluating PD-L1 may result in a wide range of different expression rates.

PIK3CA mutations in EBV-associated GC are usually accompanied by ARID1A mutations [26]. In our present study, a number of the GC cases were sequenced, and cluster analysis was performed to identify various differentially expressed genes therein. In our study, we detected a high frequency of ARID1A and PIK3CA mutations; thus in the future, we intend to investigate the relationship between ARID1A, PIK3CA and EBER, and to analyze the expression of these proteins in GC and adjacent normal tissues. Cho et al [27] found the most significantly mutated genes were TP53 (54%), ARID1A (23%), CDH1 (22%), PIK3CA (12%), RNF43 (10%) and KRAS (9%). Yoon et al [28] identified 18,377 MS mutations of five or more repeat nucleotides in gene coding sequences and untranslated regions (UTRs), and discovered 139 individual genes whose expression was downregulated in association with UTR MS mutation. In our study, numerous DDR-associated genes were detected in d-MMR patients, including ETV6, TP53, BRCA, POLE and RNF43; the most significantly mutated genes in d-MMR patients were LRP1B (79.07%), ARID1A (74.42%), RNF43 (69.77%), ZFHX3 (65.12%), TP53 (58.14%), GANS (51.16%), BRCA2(51.16%), PIK3CA (51.16%), NOTCH1 (51.16%), SMARCA4 (48.84%), ATR (46.51%), POLE (41.86%) and ATM (39.53%). We also identified that the mutation rate of LRP1B was high, reaching 79.07%. LRP1B belongs to the low-density lipoprotein (LDL) receptor gene family. LRP1B is similar to LRP1 of the LDL receptor family. Due to the interaction between these receptors and their ligands, they play a wide range of roles in normal cell functioning and development. It is capable of inhibiting tumor cell invasion and metastasis [29]. The LRP1B gene is also a novel candidate tumor suppressor that is associated with immunotherapeutic success. LRP1B mutations have also been associated with a high TMB and low patient survival rates. It has been found that nearly 40% of non-small cell lung cancer cell lines are inactivated by LRP1B alterations at the gene and transcriptional levels [30]. In the future, we aim to determine whether the expression levels of proteins encoded in association with GC are altered. Though the relationship between LRP1B mutations and survival in GC is not well understood.

ZFHX3 plays an important role in the biological clock, which if disrupted, may be detrimental to human health. Hence when mutated, ZFHX3 may influence the occurrence of cancer. ZFHX3 inhibits the proliferation of prostate cancer cells by downregulating MYC gene expression [31]. RNF43 mutation results in a frame shift that leads to the early truncation and potential inactivation of the associated protein, and as such, may be a predictor of pathogenesis [32]. Yu et al discovered a high frequency of RNF43 mutations in colorectal signet ring cell carcinoma, and that mutated RNF43 activates the Wnt pathway [33]. As with RNF43, frame shift mutations in the BRCA2 gene lead to the early truncation of the protein, and its subsequent inactivation may predict pathogenesis. BRCA2 mutations have been widely reported in breast cancer [34], but have not been extensively studied in GC. The PIK3CA Y1021C mutation is located within the PI3K/PI4K domain of the PIK3CA protein, which leads to an increase in the transformation ability of cultured cell lines [35]. GNAS R201C is located in the GTP binding region of the GNAS protein. R201C resulted in the loss of GTP enzyme activity, the continuous activation of downstream signals, cellular proliferation and tumor formation. Studies have also shown that the mutation rate of GNAS in non-ampullary duodenal adenocarcinoma is 6.5% [36]. However, GNAS mutations have been more extensively studied in tumors of the pancreatic and biliary system than in GC.

ATM mutation leads to premature truncation of the ATM protein. Due to the deletion of all known functional domains, predictive mutations result in the loss of protein function [37]. ARID1A is a subunit of the SWI/SNF chromatin remodeling complex. ARID1A mutations frequently occur in GC and are associated with poor patient prognosis, potentially because the AKT signaling pathway can be activated by the decreased expression or function of ARID1A. The levels of multiple immune markers and TMB in patients with ARID1A mutations were significantly higher than those in ARID1A wild-type patients. ARID1A defects are associated with MMR and MSI. The expression of PD-L1 in alimentary tract cancer patients with ARID1A mutations was significantly higher than that in wild-type patients [38–40]. In our study, NGS revealed a high number of ARID1A mutations in d-MMR patients, thus we intend to analyze the relationship between PD-L1 expression and ARID1A as a future research prospect. Kim et al revealed that the deletion of PTEN function was associated with high MSI and EBV-positive status. In patients with HER-2-positive GC, PTEN deletion mutations are associated with Trastuzumab resistance, and the loss of heterozygosis of this gene has been reported more frequently in GC [41, 42].

In solid tumor patients receiving immunotherapy, the median overall survival (OS) of patients with POLE/POLD1 mutations was significantly improved compared with that of non-carriers. Additionally, 26% of patients with POLE/POLD1 gene mutations also exhibited MSI-H status. After omitting these patients, OS in the mutant group remained improved; that is to say that in patients with MSS (who generally do not benefit from immunotherapy), the potential value of immunotherapy can still be determined according to POLE/POLD1 gene mutations [43]. Multivariate analysis confirmed that POLE/POLD1 mutation may be used as a novel independent index to predict immunotherapeutic value. MMR status can affect the treatment of gastric cancer, and d-MMR patients are more suitable for immunotherapy. Professor Patil’s study was centered around the expression of PD-L1 in gastric cancer and its association with CD8 in the immune microenvironment [44]. As with our own study, professor Patil used tissue microarrays for immunohistochemical staining; however, unlike our study, next generation sequencing data was not presented. We believe that our findings (such as the gene mutations detected) also have certain research and therapeutic significance for GC patients in the United States. Professor Patil analyzed 86 patients using tissue microarrays; we analyzed 2504 patients, and used larger tissue sections from postoperative specimens, not tissue microarrays. The immunohistochemical detection of four MMR proteins may be more accurate, though the tissue microarray area is very small, and may not fully represent the protein expression seen in patients. Furthermore, the d-MMR frequency in Professor Paitl’s study was 22% while the d-MMR rate in our study was 7.5%. Perhaps the positive part of the GC tissue samples had not been cut accurately (such that it was considered to be d-MMR), so that the resulting percentage was that much higher. In the future research, we will study the molecular markers of immune cells and tumor cells in the tumor microenvironment.

To the best of our knowledge, our study is the largest to investigate the pathological characteristics of GC patients in China. Using IHC, ISH and NGS, the results of this study provide a deeper understanding of GC, including MSI status, HER-2 and PD-L1 expression, TMB and gene alterations in GC patients, which offer a theoretical basis for the future clinical treatment of GC. Our future studies will aim to elucidate the mechanisms by which these mutations impact the development of GC. GC molecular typing is very important. However, due to a shortage of time, we did not analyze the relationship between genes, the survival period and staging. Statistical research in this area will be conducted in our next study. We believe that our research is of great significance for the future treatment of gastric cancer.

## Abbreviations

MSI: Microsatellite instability
TMB: Tumor mutation burden
PD-L1: Programmed death-ligand 1
ISH: In situ hybridization
DDR: DNA damage repair
